# Exposure of elementary school-aged Brazilian children to bisphenol A: association with demographic, social, and behavioral factors, and a worldwide comparison

**DOI:** 10.1038/s41598-024-67267-4

**Published:** 2024-10-17

**Authors:** Priscilla R. S. Rocha, Hadassa S. R. P. Moura, Nadyellem G. Silva, Francisco A. R. Neves, Fernando F. Sodré, Angélica A. Amato

**Affiliations:** 1 Faculty of Ceilandia, University of Brasilia, Brasília, Brazil; 2 Institute of Chemistry, University of Brasilia, Brasília, Brazil; 3 Laboratory of Molecular Pharmacology, Faculty of Health Sciences, University of Brasilia, Room B1 146/10, Campus Universitario Darcy Ribeiro, Brasília, Brazil

**Keywords:** Bisphenol A, Endocrine disruptors, Childhood, Environmental impact, Endocrine system and metabolic diseases

## Abstract

Bisphenol A (BPA) is a plasticizer used to synthesize polycarbonate plastics and epoxy resins and is well-known for its endocrine-disrupting action. BPA occurrence in the environment is widespread, and there is a growing concern regarding exposure to this chemical during childhood, given the findings indicating the long-lasting hazards associated with exposure during early life compared to adulthood. We examined urinary BPA concentrations from 319 elementary school-aged Brazilian children, using high-performance liquid chromatography coupled to high-resolution mass spectrometry. We found that urinary BPA was detectable in the majority of children, and that urinary BPA levels were higher among children with lower family income and lower maternal educational levels. BPA levels found herein were compared with those from countries with different regulation policies concerning exposure to BPA. They were similar to those reported from studies conducted in Egypt and Australia. Despite more protective regulatory policies in the European Union, they were similar or lower than those reported in European studies. Our findings indicate that exposure of Brazilian children to BPA is widespread and comparable to or even lower than that of countries with stricter regulatory policies.

## Introduction

Bisphenol A (BPA) is a plasticizer employed in the synthesis of polycarbonate plastics and epoxy resins, which are widely used for manufacturing everyday life products, such as food and beverage plastic containers, the lining of food and beverage cans, baby bottles, toys, water pipes, and other plastic materials^1^. It is found ubiquitously in the air, water, and soil due to the broad utilization of BPA-containing products and their disposal in the environment^2,3^. Hence, it is not surprising that wildlife and human exposure to BPA is widespread^4^. However, it is acknowledged that there is still much to understand about the potential impact of BPA on the diversity of ecosystems worldwide^4^.

Despite the broad presence of BPA in the environment, the most common route of overall human contact with BPA is through dietary exposure, mainly by the consumption of contaminated seafood food or the consumption of food and beverages packaged in plastic containers or cans with plastic lining, from which the chemical may migrate and contaminate them^5^. Exposure from non-food sources, such as dermal contact or inhalation, occurs at lower levels^5^.

BPA has been long known for its estrogenic properties^6^ mediated by the agonistic activity on estrogen receptors alpha and beta^7^. More recently, BPA was shown to interfere with thyroid hormone, G protein-coupled estrogen receptor, and pregnane X receptor signaling and impair multiple cellular signaling pathways^1^. Therefore, it is a well-established endocrine-disrupting chemical (EDC) linked to various hazards^1^. Exposure to BPA is associated with reproductive and developmental abnormalities, obesity and metabolic diseases, altered thyroid hormone action, neurotoxicity, and carcinogenesis in epidemiological studies^1^. Studies involving animal models have confirmed the later associations and provided the mechanistic basis for many unfavorable outcomes of BPA exposure^1^.

Early life is a critical developmental window characterized by enhanced sensitivity of biological processes to environmental stressors, such as BPA and other EDCs^8^. In addition, exposure to BPA may be higher in early life compared with adulthood due to differences in physiology, behavior, and diet^1,9^. Therefore, there is more significant concern about the unfavorable impacts of exposure to such chemicals during fetal life and early childhood. Indeed, epidemiological studies indicate that exposure to BPA, both prenatally and during childhood, is associated with an increased risk of obesity^10,11^ and neurodevelopmental disorders^10^, and its long-lasting consequences. Despite the inherent limitations of observational studies in addressing causality, their findings are reinforced by those from studies involving early life exposure to BPA in model systems^12–14^ and strongly suggest the need to limit exposure to EDCs, particularly during critical windows of development.

Limiting exposure to BPA and other EDCs requires individual and societal approaches, including translating the current scientific evidence into public awareness and knowledge and effective regulatory policies. In addition, the effectiveness of such approaches depends upon continuing efforts to improve and harmonize tests to identify EDCs and their mechanism of action and strategies to monitor and diminish exposure^15^. Monitoring ECD exposure, particularly, is critical to understand populational exposure patterns and to assess adherence to regulatory policies. In this study, we investigated exposure to BPA among elementary school-aged children from Brazil and explored whether socio-demographic and lifestyle-related factors were associated with the level of exposure. In addition, we compared exposure levels found among Brazilian children with those of other children worldwide.

## Methods

### Study design

This was a cross-sectional study conducted following the STROBE guidelines^16^. The study was approved by the Ethics Review Committee from the School of Health Sciences of the University of Brasilia (protocol number 37889314.5.0000.0030) and conducted according to the principles of the Declaration of Helsinki. All participants provided written, informed consent before participation.

### Participants and procedures

Children attending the first, second, and third year of elementary public schools in Ceilandia, Brasilia, Brazil, were eligible to participate, with no exclusion criteria. Nine public elementary schools were randomly selected. In each school, children attending the first, second, and third years were randomly selected, proportionally to the number of children enrolled in each school, using OpenEpi software (version 3.0). A total of 403 participants were selected in March 2017 and assessed through March and October 2017.

Selected children were assessed by trained health technicians to determine height, weight, and waist circumference. Nutritional status was classified according to the World Health Organization growth reference for school-aged children^17^. Parents or legal guardians completed a questionnaire containing information on birthweight, gestational age at birth, any breastfeeding, number of meals per day, exercise, daily screen time, maternal and paternal weight and height, maternal and paternal educational level, and monthly household income. Maternal and paternal nutritional status were assessed by determining the body mass index (kg/m^2^).

One first morning urine sample was collected from each participant at home, delivered to teachers in school, kept cool during transportation, and stored at − 80 °C until analysis. Urinary quantification of BPA was performed using high-performance liquid chromatography, coupled to high-resolution mass spectrometry. Our group has previously described the latter method, in addition to its performance and validation, by assessing urinary samples from 343 Brazilian children^18^. The detection limit was 0.03 ng/mL^18^, and BPA urinary concentration was adjusted for urinary creatinine excretion.

### Data analysis

We presented categorical data as frequency and continuous data as median and interquartile range since the latter were non-normally distributed, as assessed by the Shapiro–Wilk test. Urinary BPA concentrations across different categories of demographic, social and behavioral variables were compared using the Mann–Whitney test or one-way ANOVA. Multiple linear regression analysis was used to assess the association between demographic and clinical variables and urinary BPA concentration.

We compared our findings to those from previous studies by searching PubMed for publications addressing exposure levels of BPA in childhood by assessing urinary BPA levels after birth, using search terms related to BPA and childhood. We included studies with participants under 18 years, irrespective of the study’s aim and design.

Statistical significance was considered at p < 0.05, and all statistical analyses were conducted using Stata Software version 16 (StataCorp LLC, TX).

## Results

Out of 403 eligible children, 319 provided urinary samples and demographic, social, and behavioral information collected through a questionnaire and comprised the study subjects. The characteristics of the participants are described in Table 1. The median age of the participants was eight years, with a similar proportion of boys and girls. Most children had healthy weight, 21% were overweight or obese, and 19% had abdominal obesity, indicated by waist circumference above the 90th percentile. Most participants were not engaged in exercise regularly and had an average daily screen time of more than two hours. The majority of children’s mothers and fathers were overweight or obese (53.3% and 61.2%, respectively), the parental’s level of education was low, and monthly family income was less than three minimal wages for most participants.

**Table 1:** Characteristics of the study participants.

	All children	Urinary BPA (ng/mg creatinine)^a^	Statistical analysis
Sex—no. (%)			U (DF = 1, n = 319) = 11,645, p = 0.20^b^
Female	165 (51.7)	1.24 (0.5–3.53)	
Male	154 (48.3)	1.62 (0.70–3.46)	
Child age at assessment—years	8 (7–9)		F (DF = 1, 2, N = 319) = 1.902, p = 0.38^c^
6 to 7	126 (39.5)	1.42 (0.65–3.91)	
8	105 (32.9)	1.52 (0.55–3.82)	
9 or more	88 (27.6)	1.18 (0.52–2.51)	
Child nutritional status—no. (%)			U (1, n = 319) = 7745, p = 0.14^b^
Normal weight	252 (79.0)	1.33 (0.54–3.25)	
Overweight/obese	67 (21.0)	1.75 (0.71–4.08)	
Child WC percentile^a^			
< p90	300 (94.0)	1.41 (0.61–3.49)	
≥ p90	19 (6.0)	1.94 (0.57–4.37)	
Birthweight—no. (%)			F (DF = 2, N = 299) = 4.221, p = 0.12^c^
≤ 2500 g	247 (82.6)	1.92 (0.68–5.26)	
2501–3999 g	30 (10.0)	1.25 (0.57–3.21)	
≥ 4000 g	22 (7.4)	1.68 (1.02–8.82)	
Breastfeeding—no. (%)			U (1, n = 306) = 8878, p = 0.40^b^
No	86 (29.0)	1.62 (0.67–3.86)	
Yes	220 (72)	1.36 (0.55–3.25)	
Number of meals/day^a^			F (DF = 2, N = 310) = 3.770, p = 0.15^c^
2 or less	4 (1.29)	6.57 (2.75–10.60)	
3	26 (8.39)	1.41 (0.58–3.05)	
4 or more	280 (90.32)	1.41 (0.60–3.35)	
Fastfood consumption—no. (%)			F (DF = 2, N = 233) = 0.331, p = 0.84^c^
0 to 2 times/wk	205 (88.0)	1.53 (0.64–3.98)	
3 or 4 times/wk	17 (7.3)	1.05 (0.08–5.58)	
5 or more times/wk	11 (4.7)	1.67 (0.26–2.03)	
Exercise—no. (%)			U (1, n = 309) = 9165, p = 0.61^b^
No	224 (72.5)	1.37 (0.58–3.49)	
Yes	85 (27.5)	1.70 (0.68–3.45)	
Screen time in hours/day—no. (%)			U (1, n = 271) = 8499, p = 0.46^b^
≤ 2 h	115 (42.4)	1.30 (0.60–3.53)	
> 2 h	156 (57.6)	1.59 (0.62–3.66)	
Maternal nutritional status—no. (%)			U (1, n = 289) = 9700, p = 0.27^b^
Normal weight	135 (46.7)	1.38 (0.53–3.21)	
Overweight/obese	154 (53.3)	1.62 (0.71–3.81)	
Paternal nutritional status—no. (%)			U (1, n = 227) = 5851, p = 0.27^b^
Normal weight	88 (38.8)	1.46 (0.64–3.17)	
Overweight/obese	139 (61.2)	1.55 (0.64–3.98)	
Maternal education level—no. (%)			F (DF = 2, N = 292) = 9.059, p = **0.02**^c^
Less than high school	124 (42.5)	1.54 (0.64–3.37)	
High school	120 (41.1)	1.67 (0.67–3.92)	
Some college	19 (6.5)	0.85 (0.17–1.42)	
College or greater	29 (9.9)	0.95 (0.38–1.94)	
Paternal education level—no. (%)			F (DF = 2, N = 269) = 3.364, p = 0.33^c^
Less than high school	133 (49.4)	1.52 (0.55–3.98)	
High school	107 (39.8)	1.35 (0.57–3.46)	
Some college	11 (4.1)	0.64 (0.23–1.92)	
College or greater	18 (6.7)	1.18 (0.83–1.66)	
Monthly family income in minimal wages—no. (%)^d^			F (DF = 2, N = 270) = 6.771, **p = 0.03**^c^
Less than 3	222 (82.2)	1.62 (0.67–3.81)	
3 to 5	36 (13.3)	1.15 (0.54–3.12)	
More than 5	12 (4.5)	0.84 (0.32–1.24)	

DF: degrees of freedom, F: Kruskal–Wallis test statistic, N: sample size, U: Mann–Whitney test statistic.

Significant values are in bold.

^a^Median (IQR).

^b^Mann–Whitney U test.

^c^Kruskal–Wallis test.

^d^Monthly income in minimal wages in 2017, in Brazil (approximately U$ 283).

**Figure 1:**
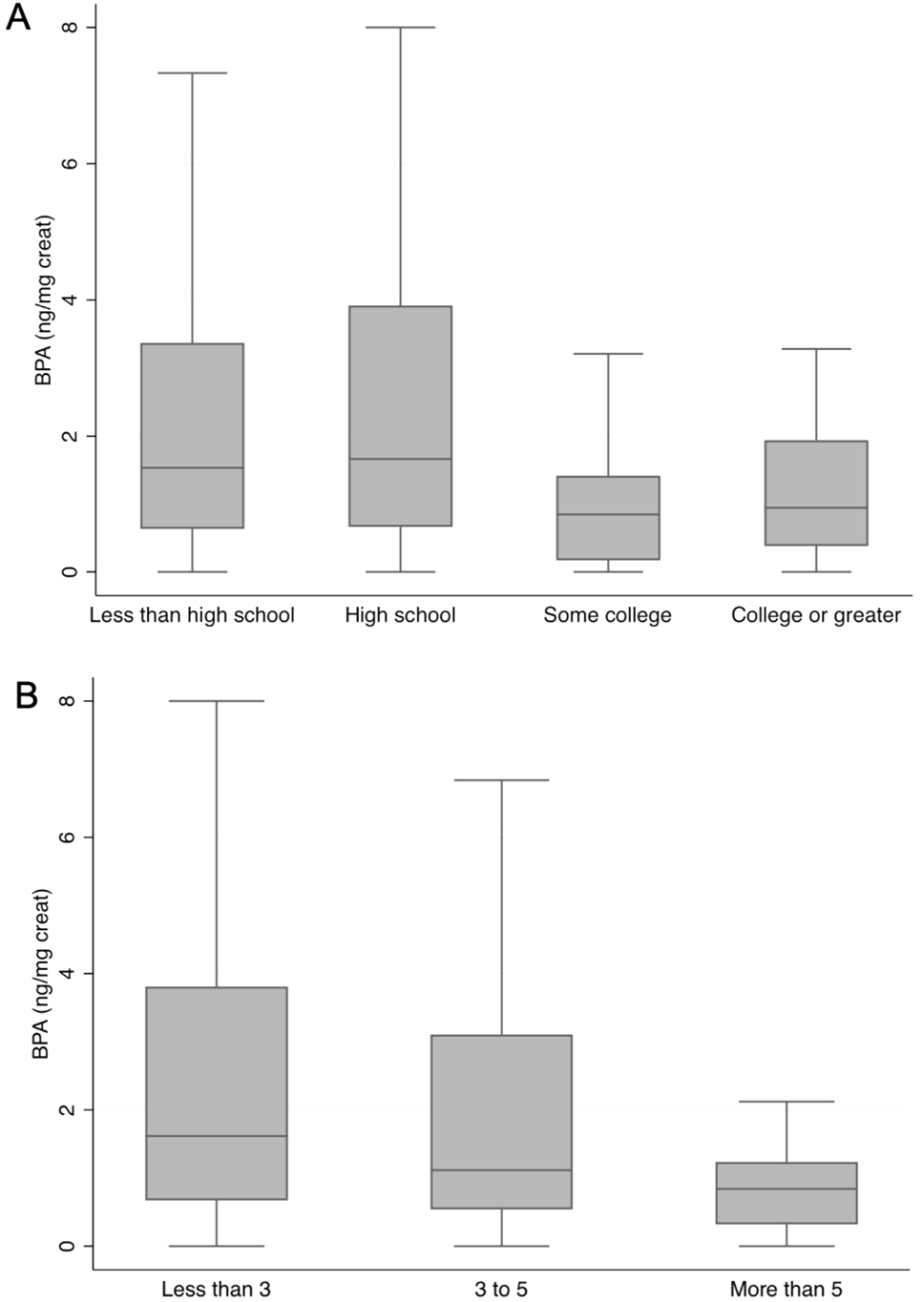
Urinary bisphenol A concentrations in elementary school-aged children according to (**A**) parents’ educational levels and (**B**) monthly family income (less than three, three to five, more than five minimal wages in 2017, in Brazil – approximately U$ 283). Data presented as box plot, n = 319.

Urinary BPA levels were above the detection limit (0.03 ng/mL) in 287 children (90%). The concentrations ranged between 0.03 and 78 ng/mL, with a median of 1.4 ng/mL (interquartile range of 0.73–3.4 ng/mL), geometric mean of 1.50 (95% confidence interval of 1.29–1.74), and a mean of 3.47 ng/mL (standard deviation of 7.05 ng/mL). Creatinine-adjusted urinary BPA concentrations were non-normally distributed and ranged between 0.08 and 250.6 ng/mg creatinine, with a median of 1.65 ng/mg creatinine (interquartile range of 0.76–3.86 ng/mg creatinine), geometric mean of 1.77 ng/mg creatinine (95% confidence interval of 1.52–2.05 ng/mg creatinine) and a mean of 5.14 ng/mg creatinine (standard deviation of 17.9 ng/mg creatinine). Creatinine-adjusted urinary BPA concentrations were significantly higher in children whose mothers had lower educational levels and those from families with lower monthly income (Table 1 and Fig. 1). In a multiple linear regression model including age, sex, maternal education level, and family income as independent variables, we found that none of the latter variables were independently associated with creatinine-adjusted urinary BPA levels (F_(7,250)_ = 1.10; n = 258; R^2^ = 0.0298; p = 0.3667; Table 2).

**Table 2:** Multiple linear regression for urinary BPA concentration (n = 258).

	β (95% CI)	p-value
Gender		
Male	1 (referent)	
Female	− 3.82 (− 8.56 to 0.92)	0.114
Age	− 1.09 (− 3.26 to 1.08)	0.323
Maternal education level		
Less than high school	1 (referent)	
High school	4.05 (− 1.14 to 9.24)	0.125
Some college	− 1.37 (− 11.21 to 8.54)	0.790
College or greater	2.07 (− 6.59 to 10.73)	0.638
Monthly family income in minimal wages		
Less than 3	1 (referent)	
3 to 5	− 3.60 (− 10.49 to 3.29)	0.304
More than 5	− 3.77 (− 15.69 to 8.16)	0.534

F_(7, 250)_ = 1.1; R^2^ = 0.0298, Adjusted R^2^ = 0.0026.

**Figure 2:**
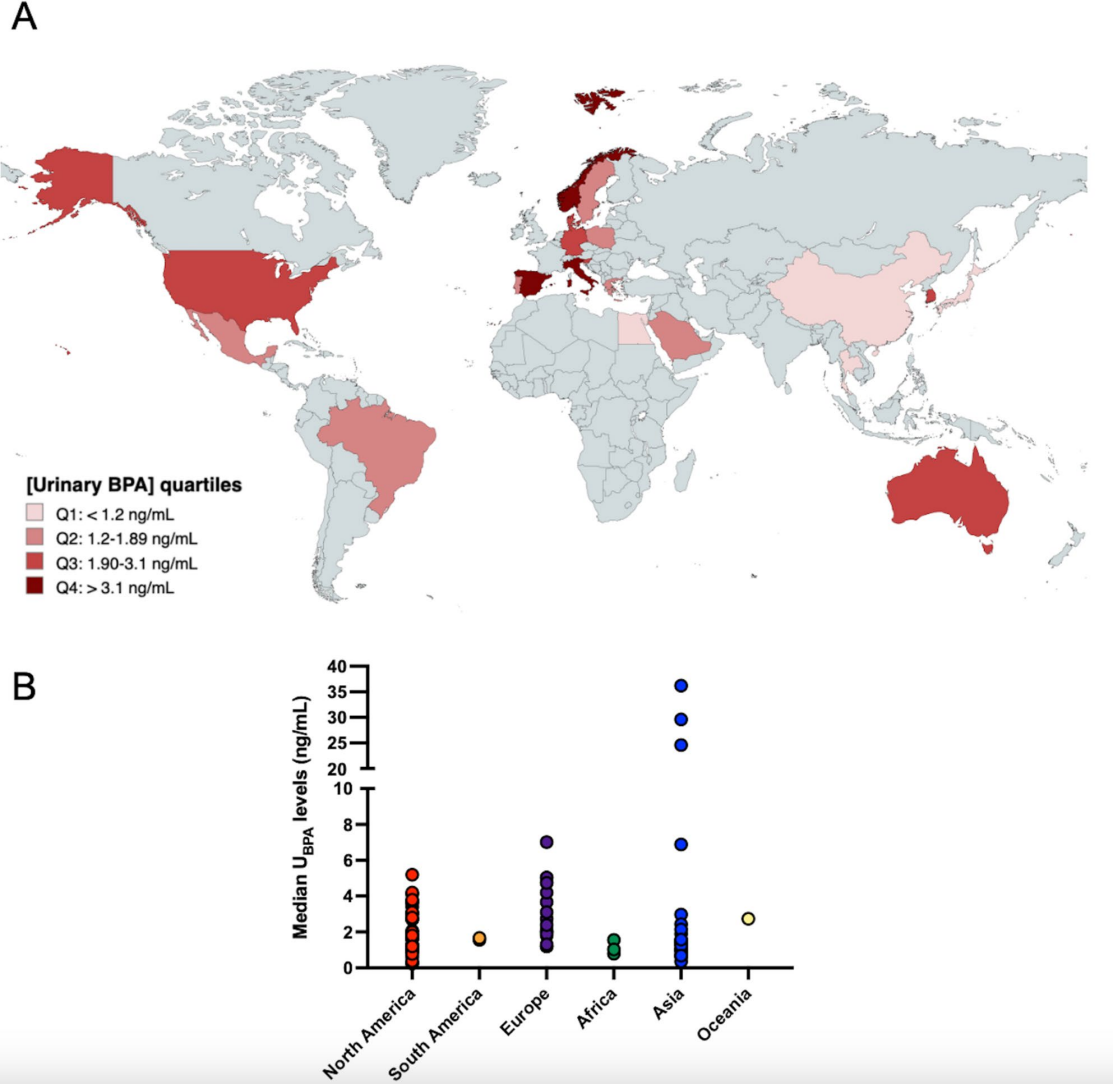
Exposure to bisphenol A during childhood assessed by urinary levels in different countries. (**A**) Exposure levels in different countries are represented according to the quartile of urinary BPA levels, which was determined by considering the results from individual studies that assessed BPA exposure in childhood. For countries in which more than one study was conducted, we calculated the median urinary BPA level considering the individual median described in each study from the country. (**B**) Median urinary bisphenol A levels according to the region from individual studies. BPA: bisphenol A. Created with MapChart (http://www.mapchart.net).

Urinary BPA detection rates varied from 87^19^ to 100%^20,21^ in North America, 82.2%^22^ to 100%^23,24^ in Europe, 47.6^25^ to 100%^26^ in Asia, and 95%^27,28^ in two related studies from Egypt, and 95%^29^ in one study from Australia. Median BPA urinary levels found in this study were similar to those reported in studies from Egypt and Australia, similar to those reported by some studies conducted in North America, Europe, and Asia, but lower than those reported by some studies from North America, Europe, and Asia (Fig. 2 and Supplementary Table 1). Interestingly, the only study we found from Iran reported geometric mean values of urinary BPA concentration considerably higher than those conducted in other countries (232 ng/mL or 282.53 ng/mg creatinine).

Notably, we found 39 studies examining BPA exposure in children aged 5 to 14 years, an age range similar to that of the participants in the current study (6 to 11 y). One study was from Africa^30^, ten from Asia^31–40^, 13 from Europe^23,24,41–51^, 14 from North America^19,52–64^, and one from South America^65^. In this age range, urinary BPA concentrations were higher in the current study than in the study conducted in Africa (median: 0.56–0.79 ng/mL), within the concentration range reported in studies conducted in Asia (mean: 0.47–2.73 ng/mL, median 0.35–6.88 ng/mL), Europe (mean: 0.47–6.92 and median 0.23–5.03 ng/mL), and similar to the previous study conducted in Brazil (median 1.66 ng/mL)^65^.

## Discussion

In this study, we found that BPA was detectable in the majority of urinary samples from 319 Brazilian children attending elementary school. Urinary BPA levels were significantly higher in children from lower-income families and whose parents had lower education levels. Moreover, BPA exposure levels in children included in this study were similar or lower to those reported in studies conducted in North America, Asia, Africa, Europe, and Australia.

The Brazilian Health Regulatory Agency established in 2011 that the migration of BPA from plastic materials and similar articles should not exceed a specific migration limit of 0.6 mg/kg of food^66^, in agreement with the Commission Regulation from the European Union^67^. In the same year, BPA was banned from feeding bottles or other articles dedicated to infant feeding in Brazil^66^. However, the effectiveness of those regulatory actions is unknown since young children may be exposed to BPA by contact with other sources such as plastic toys and other plastic food containers^5^, and biomonitoring studies to address BPA exposure in Brazil are scarce. Moreover, exposure of children aged two years or older but still experiencing early childhood is not protected against BPA exposure by current legislation. In this scenario, we investigated BPA exposure in young children by assessing urinary concentrations of this chemical and examined whether there were demographic, social, or behavioral factors that were associated with exposure.

We observed that lower family income and lower maternal education level were associated with higher urinary BPA concentration in children among all variables investigated. The finding that very few factors were associated with BPA exposure may be considered consistent with the overall widespread exposure to the chemical, independently from specific demographic, social, or behavioral variables. Additionally, education and income are long known to be correlated^26,68^. Low education levels may be related to more deficient knowledge that may negatively impact behavioral changes to reduce exposure to BPA and other ECDs, as suggested by previous studies^69^. This is reasonable since in Brazil, similarly to other countries, information on the hazards of EDCs to the general population is limited, and knowledge about those substances relies mainly on the individual’s interest and active search for the information.

We found that urinary BPA levels were higher in children with higher weight status or waist circumference, although this was not statistically significant. In some studies, exposure to BPA was associated with excess body weight in childhood^56,70–72^ but not others^39,73,74^. Interestingly, in a meta-analysis of 13 studies addressing exposure to BPA in childhood and its association with overweight, it was reported that higher BPA exposure levels significantly increased the odds of obesity when compared with lower BPA exposure, but there was no significant difference when BPA urinary levels were compared between obese and normal weight children^11^. However, it should be pointed out that the latter meta-analysis interpretation is limited since data from studies with different designs (cross-sectional and cohort) were pooled. Another meta-analysis did not report the same findings^75^.

The reasons for the inconsistent findings regarding the association between exposure to BPA and weight status in childhood between different studies are currently unclear. It may reflect differences among studies, such as statistical power to detect differences, the consideration of distinct potential confounders, and even differences in the rates of excess body weight in the population being assessed. The latter are essential questions to be addressed, given that studies indicating a positive association between exposure to BPA and obesity report an overall association of low magnitude. It is also possible that BPA may interact with other factors, such as genetics, age at exposure, diet, and sex, to affect obesity development^76^.

Moreover, given the limitations of observational studies to address causality and, in the case of cross-sectional studies, the direction of the association, it is currently not possible to establish the role of BPA and other EDCs on human obesity development^77^, or even to distinguish whether exposure to those chemicals influences weight gain or whether children with increased weight have higher exposure levels to BPA or higher BPA excretion rates. However, findings from preclinical models have consistently indicated that low-level exposure to BPA promotes increased adiposity, especially when exposure occurs at critical developmental windows^78^. Despite the limitations inherent to observational human data and of translating animal model data into human physiology, some countries have considered those data to substantiate actions for limiting human exposure to BPA following the precautionary principle.

It should be pointed out that there is considerable debate regarding the safe or acceptable levels of BPA and other EDC exposure between regulatory policymakers and the scientific community. A significant limitation of the current definition of exposure safety standards is the use of information extrapolated from traditional toxicological assessment, which is considered inaccurate in identifying EDC hazards^79^. In contrast to classical toxicological endpoints assessed after acute or chronic (2-year) exposure to increasing doses of chemicals, there is a growing body of evidence indicating that the effects of EDCs are more pronounced during critical windows of development, are non-monotonic and, hence, may be of greater magnitude at very low levels of exposure, and may be evident long periods following exposure, or even following ancestral exposure^1,76^.

We also compared exposure levels of children from different countries by searching for studies addressing exposure to BPA during childhood or adolescence and various outcomes. Most studies were conducted in North America, Europe, and Asia, and despite different regulatory policies in countries from those regions, urinary BPA detection rates in children were similar. Notably, a few studies in some Asian countries, such as China and Taiwan, reported lower exposure rates, although exposure levels were high in some studies from Taiwan. Moreover, the level of exposure, assessed by mean, geometric mean, median, or range of BPA urinary concentrations reported by studies conducted in different countries, were overall similar. This is an interesting finding, considering that regulatory policies regarding exposure to BPA vary between countries, as reviewed elsewhere^15^.

Regulatory policies are most advanced in the European Union and the United States. In the European Union, specifically, policies may be viewed as more protective since they embrace the precautionary principle and intent on minimizing overall exposure to BPA and other EDCs but focusing on critical developmental windows by following a hazard-based approach, in which evidence of potential hazard is enough to substantiate actions to limit exposure^80,81^. In contrast, in the United States, regulatory policies follow a risk-based approach, which requires direct human evidence of adverse effects^82,83^. Nevertheless, exposure levels during childhood were similar when comparing studies conducted in the United States and European countries, suggesting that further action is required to translate regulatory policies into protecting children from exposure to BPA. Chemical pollution is a global threat comparable to climate change and biodiversity loss concerning human and environmental health. Therefore, it would be possibly best addressed by scientific knowledge-based intergovernmental agreements^84^, following the ‘one planet: one health’ approach recently proposed by environmental scientists^85^.

Our study is the second assessment of urinary BPA levels in Brazilian children and indicating broad exposure to this chemical. Exposure was higher among children whose parents had lower educational levels and whose families had lower income but was not associated with other demographic, social, or behavioral factors. A comparison with other countries with different regulatory policies concerning exposure to BPA indicated similar BPA detection rates in urine. However, exposure levels found herein were more similar to those reported in studies from Egypt and Australia and lower than those reported in studies conducted in North America, Europe, and Asia.

## Supplementary Information

Supplementary Information.

## Data Availability

Individual deidentified participant data will be provided upon request (angelicamato@unb.br).
