# Supplementary material for: Exposure of elementary school-aged Brazilian children to bisphenol A: association with demographic, social, and behavioral factors, and a worldwide comparison
Source: Sci Rep. 2024 Oct 17;14:24355. doi: 10.1038/s41598-024-67267-4 (PMC11487177; doi:10.1038/s41598-024-67267-4)
Supplement: Supplementary file 1 — Supplementary Information. [file 41598_2024_67267_MOESM1_ESM.docx]

Supplementary Table 1. Exposure to bisphenol A in childhood from studies conducted in different world regions.

|  | **Study objective** | **Children’s age**  **and sample size** | **Detection method** | **Detection**  **rate (%)** | **[Urinary BPA]**  **Mean or median ^a^** | **[Urinary BPA]**  **Other measures ^b^** |
| --- | --- | --- | --- | --- | --- | --- |
| **Africa** |  |  |  |  |  |  |
| Nahar et al., 2012 and Kim et al., 2013 ^1,2^  Egypt | To assess the exposure of girls to BPA and its determinants | 10-13 y  N = 60 | HPLC-MS/MS | 95 | Mean: 1.75 ng/mL  Median: 1.02 ng/mL | GM: 1 ng/mL  GSE: 0.4 ng/mL  Range < LOD-10.8 ng/mL |
| Youssef et al., 2018 ^3^  Egypt | To compare urinary BPA between children with asthma and healthy controls | 3-8 y  N = 97 | HPLC-MS/MS | NR | Median  Control: 0.79 ng/mL  Asthmatic children: 0.56 ng/mL | Control group  Median:  Asthmatic children  Median: 1 |
| **Asia** |  |  |  |  |  |  |
| Wang et al., 2012 ^4^  China | To investigate the association between BPA exposure and BMI | 8-15 y  N = 259 | UPLC-MS/MS | 84.9 | Median: 0.60 ng/mL | IQR: 0.20-1.37 ng/mL  GM: 0.45 ng/mL (95%CI: 0.37-0.55)  Range: 0.05-16.3 ng/mL |
| Li et al., 2013 ^5^  China | To investigate exposure to BPA and its association with type of drinking bottles | 3-24 y  N = 287 | GC-MS | 100 | - | GM: 2.75 ng/mg creatinine  GSD: 2.00 ng/mg creatinine  Range: 0.41-198.05 ng/mg creatinine |
| Wang et al., 2014 ^6^  China | To investigate exposure to BPA and its association with age and body mass | 9-12 y  666 | UPLC-MS/MS | 98.9 | Mean: 2.8 ng/mL  Median: 1.00 ng/mL | Median: 2.2 ng/mg creatinine  IQR: 0.6-2.2 ng/mL / 0.99-5.15 ng/mg creatinine  Mean: 7.22 ng/mg creatinine  SD: 14.2 ng/mL / 34.63 ng/mg creatinine  GM 1.11 ng/mL (95% CI 1.02-1.21) / 2.32 ng/mg creatinine (95%CI: 2.1-2.56)  Range: 0.1-326.0 ng/mL / <LOD-785.39 ng/mg creatinine |
| Zhang et al., 2015 ^7^  China | To investigate exposure to BPA | 8-10 y  N = 256 | LC- MS/MS | 99.2 | Mean: 2.47. ng/mL | Mean: 1.93 ng/mg creatinine  GM: 1.55 ng/mL / 2.52 ng/mg creatinine  Max: 24.9 ng/mL / 74.3 ng/mg creatinine |
| Lv et al., 2016 ^8^  China | To investigate the association between exposure to BPA and oxidative DNA damage | 3-6 y  N = 96 | HPLC–MS/MS | 87 | Median: 1.44 ng/mL | Median: 6.48 ng/mg creatinine  GM: 1.08: ng/mL / 4.61 ng/mg creatinine  GSD: 4.17 ng/mL / 14.8 ng/mg creatinine  Range: <LOD-37.1 ng/mL / < LOD-119 ng/mg creatinine |
| Chen et al., 2018 ^9^  China | To investigate the association between exposure to BPA and ICPP in girls | 6-9 y  N=272 | HPLC/FLD | Control: 58.5  ICPP: 83.8 | Median  Control: 1.02 ng/mL  ICCP: 6.88 ng/mL | Control  Median: 1.17 ng/mg creatinine  IQR: < LOD-3.58 ng/mL / < LOD-4.24 ng/mg creatinine  Range: < LOD-15.73 ng/mL / <LOD-44.8 ng/mg creatinine  ICCP  Median: 6.35 ng/mg creatinine  IQR: 1.92-12.86 ng/mL / 2.3-10.85 ng/mg creatinine  Range: < LOD-40.73 ng/mL / <LOD-61.55 ng/mg creatinine |
| Chen et al., 2018 ^10^  China | To investigate exposure to BPA and understand the potential sources of BPA exposure | 8-11 y  N = 283 | HPLC-MS/MS | 93 | Mean: 0.84 ng/mL  Median: 0.35 ng/mL | SD: 2.12 ng/mL  Range: < LOD-31.1 ng/mL |
| Li et al. 2018 ^11^  China | To investigate the association between exposure to BPA and altered behavior | 6-12 y  N = 465 | HPLC-MS/MS | 90.8 | Median: 2.97 ng/mL | Median: 6.81 ng/mg creatinine  IQR: 1.17-5.83 ng/mL / 3.22-18.2 ng/mg creatinine  GM: 2.36 ng/mL / 2.71 ng/mg creatinine  GSD: 6.08 ng/mL / 9.25 ng/mg creatinine  Range: < LOD-58.9 ng/mL / <LOD-122 ng/mg creatinine |
| Wang et al., 2019 ^12^  China | To investigate the association between exposure to BPA and pubertal height growth | 9-18 y  N = 754 | HPLC/FLD | NR | - | GM: 1.6 ng/mL (95%CI: 1.4-1.8) / 1.2 ng/mg creatinine (95%CI: 1-1.3) |
| Guo et al., 2020 ^13^  China | To investigate the association between exposure to BPA and obesity | 3 y  N = 498  7 y  N = 454 | GC-MS/MS | 93 | Median  3 y: 1.04 ng/mL  7 y: 1.41 ng/mL | 3 y  Median: 2.59 ng/mg creatinine  Range: 0.19-22.9 ng/mL / 0.4-38.1 ng/mg creatinine  7 y  Median: 2.41 ng/mg creatinine  Range: <LOD-770 ng/mL / <LOD-766 ng/mg creatinine |
| Guo et al., 2020 ^14^  China | To investigate the association between exposure to BPA and thyroid function/behavior | 10 y  N = 386 | GC-MS/MS | 95.1 | Median: 1.29 ng/mL | Median: 1.34 ng/mg creatinine  IQR: 0.56-2.4 ng/mL / 0.78-2.51 ng/mg creatinine  Range < LOD-440 ng/mL / < LOD-329 ng/mg creatinine |
| Yang et al., 2022 ^15^  China | To investigate exposure to BPA | 0.75-6 y  N = 46 | UPLC-MS/MS | 100 | Median: 2.43 ng/mL | GM: 3.05 ng/mL  Range: 0.24-38.8 ng/mL |
| Chen et al., 2023 ^16^  China | To investigate the association between exposure to BPA and obesity | 7 y  N = 456 | HPLC–MS/MS | 84.7 | Median: 0.72 ng/mL | Median: 1.45 ng/mg creatinine  Range < LOD-42.31 ng/mL / < LOD-131.44 ng/mg creatinine |
| Xue et al., 2015 ^17^  India | To compare urinary BPA between children with obesity and normal-weight controls | 2-14 y  N = 76 | ESI-MS/MS | 99 | Mean: 7.43 ng/mL | Mean: 8.63 ng/mg creatinine  GM: 5.08 ng/mL / 2.42 ng/mg creatinine  GSD: 7.02 ng/mL / 35.2 ng/mg creatinine  Range: 0.07-41.4 ng/mL / 0.05-305 ng/mg creatinine |
| Amin et al., 2019 ^18^  Iran | To investigate the association between exposure to BPA and obesity/cardiometabolic risk factors | 6-18 y  N = 132 | GC-MS/MS | 100 | - | GM: 232.6 ng/mL / 282.53 ng/mg creatinine  GSD 126.05 ng/mL / 166.02 ng/mg creatinine |
| Gys et al., 2020 ^19^  Hokkaido Study on Environment and Children’s Health  Japan | To investigate exposure to BPA and its temporal trends | 7 y  N = 396 | GC-MS/MS | 89 | Median: 0.89 ng/mL | IQR: 0.52-1.66 ng/mL  Range: < LOD-14.44 ng/mL |
| Al-Daghri et al., 2017 ^20^  Saudi Arabia | To investigate the association between exposure to BPA and metabolic disturbances | 13-16 y  N = 177 | GC-MS/MS | 98 | Median  Non-obese: 1.89 ng/mL  Obese: 1.54 ng/mL | Non obese children  IQR: 1.32-2.62 ng/mL  Obese children  IQR: 1.07-2.44 ng/mL |
| Hong et al., 2013 ^21^  South Korea | To investigate the association between exposure to BPA and behavior/learning | 8-11 y  N = 1,089 | HPLC-ESI-MS/MS | 100 | Median: 1.23 ng/mL | Median: 1.28 ng/mg creatinine  IQR: 0.67-2.29 ng/mL / 0.76-2.18 ng/mg creatinine  GM: 1.32 ng/mg creatinine  GSD: 2.32 ng/mg creatinine  Range: 0.16-125.16 ng/mL / 0.14-300.15 ng/mg creatinine |
| Jung et al., 2022 ^22^  KoNEHS 2015-2017  South Korea | To investigate exposure to BPA | 3-18 y  N = 2,380 | UPLC-MS/MS | 3-5 y: 98.87  6-11 y: 96.05  12-18 y: 96.9 | 3-5 y  Mean: 45.9 ng/mL  Median 34.6 ng/mL  6-11 y  Mean: 37.5 ng/mL  Median 29.6 ng/mL  12-18 y  Mean: 19.9 ng/mL  Median: 24.6 ng/mL | 3-5 y  Median 95%CI: 21.3-58.7 ng/mL  GM: 34.6ng/mL (95%CI: 31.3-38.2)  6-11 y  Median 95%CI: 18.7-47.2 ng/mL  GM 28.8 ng/mL (95%CI: 26.5-31.2)  12-18 y  Median 95%CI: 14.8-24.6 ng/mL  GM 13.6 ng/mL (95%CI: 12.0-15.5) |
| Choi et al., 2014 ^23^  South Korea | To investigate the association between exposure to BPA and obesity in girls | 6-14 y  N = 127 | GC/MS | NR | Mean: 2.72-3.66 ng/mL | SD: 1.82-3.23 ng/mL  Range: <LOD-18.73 ng/mL |
| Lim et al., 2017 ^24^  EDC Study  South Korea | To investigate the association between exposure to BPA and social impairment | 4 y  N = 304 | HPLC–MS/MS | NR | Mean: 3.2 ng/mL | Mean: 4.9 ng/mg creatinine  SD: 5 ng/mL / 10.8 ng/mg creatinine |
| Jang et al., 2021 ^25^  EDC Study  South Korea | To investigate the association between exposure to BPA and thyroid hormone levels | 6 y  N = 574 | HPLC–MS/MS | 98.3 | Mean: 2.73 ng/mL | SD: 7.15 ng/mL |
| Hwang et al., 2022 ^26^  KoNEHS 2015-2017  South Korea | To investigate the association between exposure to BPA and allergic outcomes | 3-11 y  N = 1,458 | UPLC-MS/MS | 97 | - | Preschool children  GM: 2.41 ng/mL (95%CI: 2.05-2.83)  School children  GM: 1.70 ng/mL (95%CI: 1.49-1.95) |
| Kim et al., 2022 ^27^  KoNEHS 2015-2017  South Korea | To investigate the association between exposure to BPA and metabolic outcomes | 3-18 y  N = 1,454 | GC-MS | 97.31 | 3-12 y  Median: 2.14 ng/mL  13-18 y  Median: 1.48 ng/mL | 3-12 y  IQR: 1.08-3.95 ng/mL  13-18 y  IQR: 0.76-3.11 ng/mL |
| Kim et al., 2022 ^28^  EDC study  South Korea | To investigate the association between exposure to BPA and ADHD | 4 to 8 y  N = 619 | GC-MS/MS | 97 | - | 4 y  GM: 3.29 ng/mg creatinine  GSD: 2.51 ng/mg creatinine  6 y  GM: 2.36 ng/mg creatinine  GSD: 2.32 ng/mg creatinine  8 y  GM: 1.96 ng/mg creatinine  GSD: 2.58 ng/mg creatinine |
| Lee et al., 2022 ^29^  EDC Study  South Korea | To investigate the association between exposure to BPA and serum uric acid | 6 y  N = 489 | UPLC-MS/MS | 99.9 | Median: 1.582 ng/mL | Range: 0.15-153.126 ng/mL  IQR: 0.992-2.503 ng/mL  GM: 1.629 ng/mL  GSD: 2.452 ng/mL |
| Wang et al., 2016 ^30^  Childhood Environment and Allergic Diseases Study  Taiwan | To investigate the association between exposure to BPA and atopic disorders | 3-6 y  200 | UPLC-MS/MS | NR | - | 3 y  GM: 11.84 ng/mL  GSD: 3.35 ng/mL  6 y  GM: 8.84 ng/mL  GSD: 2.57 ng/mL |
| Chang et al., 2017 ^31^  Taiwan | To investigate exposure to BPA and its association with age and sex | 51 children and 71 adolescents | HPLC-MS/MS | 47.6 | - | GM: 5.81 ng/mL  Range: 1.94-190.10 ng/mL |
| Lin et al., 2018 ^32^  Taiwan | To investigate the association between exposure to BPA and GSTP1 polymorphisms | 3 y  N = 453 | UPLC-MS/MS | NR | - | Group 1  GM: 14.7 ng/mL  GSD 1.07 ng/mL  Group 2  GM:10.93 ng/mL  GSD: 1.12 ng/mL |
| Pornkunwilai et al., 2015 ^33^  Thailand | To investigate the association between exposure to BPA obesity | 3-18 y  N = 376 | HPLC–MS/MS | 75.3 | Median: 0.68 ng/mL | Median: 0.53 ng/mg creatinine  IQR: 0.06-1.48 ng/mL / 0.04-1.12 ng/mg creatinine  Range: <LOD-8.75 ng/mL |
| Tosirisuk et al., 2022 ^34^  Thailand | To investigate exposure to BPA in children with type 1 diabetes | 3-25 y  N = 75 (T1D), 113 (controls) | HPLC | NR | - | Control  Median: 10.13 ng/mg creatinine  IQR: 0-54.01 ng/mg creatinine  T1D  Median: 31.5 ng/mg creatinine  IQR: 7.87-64.45 ng/mg creatinine |
| Çok et al., 2020 ^35^  Turkey | To investigate exposure to BPA | 3-6 y  N = 125 | HPLC–MS/MS | 76.8 | - | Mean 1.79 ng/mg creatinine  Range: < LOD-18.36 ng/mg creatinine |
| **Europe** |  |  |  |  |  |  |
| Covaci et al., 2015 ^36^  Belgium, Denmark, Luxembourg, Slovenia, Spain and Sweden  COPHES/DEMOCOPHES consortium | To investigate exposure to BPA and its determinants | 5-12 y  N = 653 | LC-MS/MS and GC-ECNI/MS | 91.1 | Median: 1.96 ng/mL | IQR: 1.05-3.76 ng/mL  GM 1.97 ng/mL (95%CI: 1.81-2.15)  Range: < LOD-821.9 ng/mL |
| Koppen et al., 2019 ^37^  COPHES/DEMOCOPHES consortium  Belgium | To investigate exposure to BPA and its determinants | Median 8 y  N = 129 | GC-ECNI/MS | > 92.8 | - | GM: 2.35-2.55 μg/L |
| Frederiksen et al., 2013 ^38^  Copenhagen Puberty Study 2006–2008  Denmark | To investigate exposure to BPA and its association with age and sex | 6-16 y  N = 129 | LC-MS/MS | 82.2 | Mean: 3.61 ng/mL  Median: 2.06 ng/mL | IQR: 0.73-3.31 ng/mL  Range: <LOD-130 ng/mL |
| Becker et al., 2009 ^39^  The German Environmental Surveys  Germany | To investigate exposure to BPA | 3-14 y  N = 599 | GC-MS/MS | 98.7 | Mean: 2.7 ng/L  Median: 2.74 ng/mL | GM: 2.66 ng/mL (95%CI 2.44-2.89)  Maximum: 205 ng/mL |
| Kasper-Sonnenberg et al., 2014 ^40^  Germany  Duisburg Birth Cohort and Bochum Cohort Studies | To investigate exposure to BPA and its association with age | 8-10 y  N = 465 | LC/LC–MS/MS | 100 | Median: 2.04 ng/mL | Median: 1.76 ng/mg creatinine  GM 2.2 μg/L (95%CI 2.03-2.39) / 1.97 μg/g creatinine (1.82-2.14) |
| Kasper-Sonnenberg et al., 2017 ^41^  Germany | To investigate the association between exposure to BPA pubertal development | 8-13 y  N = 472 | LC/LC-MS/MS | NR | - | Boys  GM: 2.1 ng/mL (95%CI: 1.9-2.3)  Girls  GM: 2.1 ng/mL (95%CI: 1.9-2.5) |
| Myridakis et al., 2015 ^42^  ‘Rhea’ Project  Greece | To investigate exposure to BPA | 2 y  N = 239 | LC-MS | 99.6 | Median: 1.2 ng/mL  Mean: 2.6 ng/mL | Median: 1.1 ng/mg creatinine  Mean: 2.4 ng/mg creatinine  GM: 1.2 ng/mL (95% CI 1.1-1.4) / 1.2 ng/mg creatinine (95%CI 1.1-1.4)  95%CI 1.1-1.4 (1.1-1.4)  Range: < LOD-144 ng/mL / 116.1 ng/mg creatinine |
| Vafeiadi et al., 2016 ^43^  Greece | To investigate the association between exposure to BPA and obesity/cardiometabolic traits | 2.5 y (N = 235)  4 y (N = 500) | HPLC-ESI-MS/MS | NR | Median  2.5 y: 2.0 ng/mL  4 y: 1.2 ng/mL | 2.5 y  GM: 2.0 ng/mL (95%CI: 1.7-2.4) / 5.1 ng/mg creatinine (95%CI 4.4-5.9)  Median: 5.1 ng/mg creatinine  IQR: 0.9-5.2 ng/mL / 2.2-11.4 ng/mg creatinine  Range: <LOD-68.6 ng/mL / 0.2-121.7 ng/mg creatinine  4y  GM: 1.1 ng/mL (95%CI: 1.0-1.2) / 1.9 ng/mg creatinine (95%CI 1.7-2.0)  Median: 1.9 ng/mg creatinine  IQR: 0.6-2.1 ng/mL / 1.0-3.4 ng/mg creatinine  Range: <LOD-59.2 ng/mL / 0.1-67.3 ng/mg creatinine |
| Nicolucci et al., 2013 ^44^  Italy | To validate a method for determination of urinary BPA | 6-14 y  N = 105 | LC/ESI-MS/MS | 95 | Mean  Normal weight: 0.47 ng/mL  Obese: 0.72 ng/mL | Range: 0.2-1.45 ng/mL  Normal weight  SD 0.12 ng/mL  Obese  SD 0.23 ng/mL |
| D’Aniello et al., 2015 ^45^  Italy | To investigate the association between exposure to BPA and obesity | 4-15 y  N = 54 | GC-MS/MS | 94.4 | Mean: 1.13 ng/mL | Mean: 0.91 ng/mg creatinine  SD: 0.89 ng/mL / 0.74 ng/mg creatinine  Range: < 0.01-2.71 ng/mL |
| Bellisario et al., 2021 ^46^  Italy | To investigate exposure to BPA in neonates and its determinants | Neonates  N = 134 | UPLC-ESI-MS/MS | NR | Mean: 0.13 ng/mL | Mean: 0.48 ng/mg creatinine  SD: 0.3 ng/mL / 1.13 ng/mg creatinine  Range: 0.02-0.74 ng/mL / 0.02-2.5 ng/mg creatinine |
| Tait et al., 2021 ^47^  Italy | To investigate exposure to BPA | 4-14 y  N = 900 | GC-MS/MS | 96.1 | Median 7.02 ng/mL | Median: 6.77 ng/mg creatinine  IQR: 4.16-12.06 ng/mL / 3.92-12.77 ng/mg creatinine  GM 7.06 ng/mL (95%CI 6.57-7.6) / 6.77 ng/mg creatinine (95%CI 3.92-12.77) |
| Sakhi et al., 2018 ^48^  Norway | To investigate exposure to BPA and its determinants | 6-12 y  N = 54 | UPLC-MS-MS | 100 | Mean: 4.54 ng/mL  Median: 3.67 ng/mL | IQR: 2.62-4.85 ng/mL  GM: 3.70 ng/mL  Range: 1.46 to 36.4 ng/mL |
| Gari et al., 2021 ^49^  REPRO-PL  Poland | To investigate the association between exposure to BPA and neurodevelopmental outcomes | 7 y  N = 250 | HPLC-MS/MS | 99.6 | Median: 1.8 ng/mL | IQR: 1.1-2.8 μg/L  GM: 1.9 μg/L (95%CI: 1.7-2.1)  Range: < LOD-53.1 μg/L |
| Correia-Sá et al., 2017 ^50^  Portugal | To investigate exposure to BPA and its determinants | 4-18 y  N = 110 | HPLC-MS/MS | 91 | Mean 4.11 ng/mL  Median: 1.89 ng/mL | Mean: 3.6 ng/mg creatinine  SD: 8.91 ng/mL / 6.17 ng/mg creatinine  Median: 1.92 ng/mg creatinine  GM: 1.58 ng/mL (95%CI: 1.18-2.13) / 1.76 ng/mg creatinine (95%CI: 1.38-2.41)  Maximum: 66.9 ng/mL / 46 ng/mg creatinine |
| Casas et al., 2011 ^51^  INMA  Spain | To investigate exposure to BPA and its determinants | 4 y  N = 30 | HPLC-MS/MS | 96.7 | Median: 4.2 ng/mL | IQR: 5.5 ng/mL |
| Casas et al., 2013 ^52^  INMA  Spain | To investigate exposure to BPA and its determinants | 4 y  N = 130 | LC-MS | 100 | Median: 3.1 ng/mL | Median: 3.7 ng/mg creatinine  IQR: 1.7-4.8 μg/L / 2.5-5.3 μg/g creatinine  GM: 3.1 ng/mL / 3.9 ng/mg creatinine  GSD: 2.2 ng/mL / 2.0 ng/mg creatinine  Range: 0.5-33.3 ng/mL / 1.1-36.2 ng/mg creatinine |
| Cutanda et al., 2015 ^53^  Spain | To investigate exposure to BPA | 6-11 y  N = 120 | LC/LC-MS/MS | 98.3 | - | GM: 2.01 ng/mg creatinine (95%CI: 1.66-2.43) |
| Perez-Lobato et al., 2016 ^54^  Spain | To investigate the association between exposure to BPA and behavior | 9-11 y  N = 300 | LC-MS-MS | 100 | Mean: 6.92 ng/mL  Median: 4.76 ng/mL | Mean: 7.97 ng/mg creatinine  SD: 7.54 ng/mL / 9.37 ng/mg creatinine  Range: 0.25-62.4 ng/mL / 0.26-76.3 ng/mg creatinine  Median: 4.75 .97 ng/mg creatinine  IQR: 2.77-9.03 ng/mL / 2.75-10.23 ng/mg creatinine  GM: 4.58 ng/mL / 5.07 ng/mg creatinine  GSD: 0.95 ng/mL / 0.95 ng/mg creatinine |
| Mustieles et al., 2018 ^55^  INMA  Spain | To investigate the association between exposure to BPA and reproductive hormones/cortisol in peripubertal boys | 9-11 y  N = 172 | LC-MS |  | Median: 5.03 ng/mL | GM: 5.1 ng/mL / 5.8 ng/mg creatinine  GSD: 1.07 ng/mL / 1.09 ng/mg creatinine  Range: 0.25-44.4 ng/mL / 0.46-63.7 ng/mg creatinine  Median: 5.37 ng/mg creatinine  IQR: 3.06-9.57 ng/mL / 3.2-11.0 ng/mg creatinine |
| Mustieles et al., 2019 ^56^  Spain | To investigate the association between exposure to BPA and adiposity measures in peripubertal boys | 9-11 y  N = 298 | LC-MS | 100 | Median 4.74 ng/mL | IQR: 2.86-8.96 ng/mL  Range: 0.25-62.4 ng/mL |
| Tratnik et al., 2019 ^57^  Slovenia | To investigate exposure to BPA and its determinants | 6-11 y  N = 145 | GC-MS/MS | 88 | Median: 2.39 ng/mL | Median: 1.86 ng/mg creatinine  IQR: 0.67-4.57 ng/mL / 0.58-3.84 ng/mg creatinine  GM: 1.02 ng/mL (95%CI: 1.42-2.31) / 1.51 ng/mg creatinine (95%CI: 1.18-1.92)  Range: <LOD-69.8 ng/mL / <LOD-25.8 ng/mg creatinine |
| Larsson et al., 2014 ^58^  COPHES/DEMOCOPHES consortium  Sweden | To investigate exposure to BPA and its determinants | 6-11 y  N = 97 | LC-MS/MS | 100 | Median: 1.31 ng/mL | Median: 1.46 ng/mg creatinine  IQR: 0.9-6.24 ng/mL / 1.0-6.3 ng/mg creatinine  GM: 1.48 ng/mL (95%CI: 1.27-1.73) / 1.67 ng/mg creatinine (95%CI 1.43-1.95) |
| **America** |  |  |  |  |  |  |
| Arbuckle et al., 2016 ^59^  CHMS  Canada | To investigate the association between exposure to BPA and learning/behavioral problems | 6-11 y  N = 1,038 | GC-MS-MS | 93.6 | - | GM: 1.31 μg/L  p95: 7.24 μg/L |
| England-Mason et al., 2021 ^60^  APrON cohort  Canada | To investigate the association between exposure to BPA and executive function difficulties | 3 mo  N = 302 | HPLC-Orbitrap MS | 89.1 | Mean: 1.76 ng/mL | Mean: 1.0 ng/mg creatinine  SD: 0.24 ng/mL / 0.22 ng/mg creatinine  GM 0.93 ng/mL / 1.11 ng/mg creatinine  Range: 0.17-54.64 ng/mL / 0.18-41.62 ng/mg creatinine |
| Findlay et al., 2015 ^61^  CHMS  Canada | To investigate the association between exposure to BPA and behavioral outcomes | 6-17 y  N = 2,730 | GC-MS/MS | 93.6 | - | GM: 1.3 ng/mL (95%CI 1.1-1.4) |
| Grohs et al., 2019 ^62^  APrON study  Canada | To investigate the association between exposure to BPA and brain structure/behavior | 3-4 y  N = 98 | LC-Orbitrap MS | 91 | Mean: 1.0 ng/mL | Mean: 1.0 ng/mL  Median: 0.9 ng/mL  IQR: 0.4-1.8 ng/mL  GM: 1.0 ng/mL (95%CI: 0.6-1.5)  Max 10.9 ng/mL |
| Jacobson et al., 2020 ^63^  Chronic Kidney Disease in Children study  US and Canada | To investigate the association between exposure to BPA and kidney function in children with CKD | 6 mo and 11 y  N = 618 | HPLC-ESI-MS/MS | 78.3 | Median: 0.60 ng/mL | IQR: 0.22-1.42 ng/mL  GM: 0.59 ng/mL  GSD: 3.49 ng/mL |
| Lewis et al., 2013 ^64^  ELEMENT project  Mexico | To investigate exposure to BPA and its determinants | 8-13 y  N = 108 | ID-LC-MS/MS | Boys: 89  Girls: 87 | Median  Boys: 1.2 ng/mL  Girls: 1.1 ng.mL | Boys  IQR: <LOD-1.9 ng/mL  GM 1.1 ng/mL  Girls  IQR: 0.6-2.2 ng/mL  GM: 1.2 ng/mL |
| Ferguson et al., 2014 ^65^  ELEMENT project  Mexico | To investigate the association between exposure to BPA and sex hormones/puberty in boys | 8-14 y  N = 114 | HPLC-MS/MS | NR | Median: 2.06 ng/mL | IQR: 1.38-3.14 ng/mL  GM: 1.30 ng/mL  GSD: 2.24 ng/mL  Maximum: 22.2 ng/mL |
| Watkins et al., 2014 ^66^  Mexico City birth cohort  Mexico | To investigate the association between exposure to BPA and sexual maturation in girls | 8-19 y  N = 129 | ID-LC-MS/MS | NR | Median: 1.3 ng/mL | IQR: 0.583-2.57 ng/mL  GM: 1.2 ng/mL  Range: < LOD-5.82 ng/mL |
| Calafat et al., 2008 ^67^  NHANES 2003-2004  US | To investigate exposure to BPA and its determinants | 6-19 y  N = 2,517 | HPLC-MS/MS | 92.6 | Median  6-11 y: 3.7 ng/mL  12-19 y: 4.2 ng/mL | 6-11 y  Median: 4.2 ng/mg creatinine  IQR: 1.7–6.7 ng/mL / 2.7–7.1 ng/mg creatinine  GM: 3.6 ng/mL (95%CI: 2.9-4.3) / 4.3 ng/mg creatinine (95%CI: 3.6–5.1)  12-19 y  Median: 2.7 ng/mg creatinine  IQR: 1.9-7.5 ng/mL / 1.7-34.7 ng/mg creatinine  GM: 3.7 ng/mL (95%CI: 3.3-4.2) / 2.8 ng/mg creatinine (95%CI: 2.5-3.1) |
| Teitelbaum et al., 2008 ^68^  US (New York) | To investigate the temporal variability of urinary BPA among minority children | 6-10 y  N = 35 | HPLC-MS/MS | 95 | - | 1^st^ tertile: 1.6 to 2.6 ng/mL  2^nd^ tertile: 2.6 to 3.9 ng/mL  3^rd^ tertile: 3.7 to 8.1 ng/mL |
| Wolff et al., 2008 ^69^  US (New York) | To investigate the association between exposure to BPA and pubertal status in girls | 9 y  192 | NR | NR | - | GM: 0.11-0.24 μg/g creatinine  GSD: 10.3-12.9 μg/g creatinine |
| Wolff et al., 2010 ^70^  BCERC  2004-2007  US (New York, Cincinnati, Ohio, California) | To investigate the association between exposure to BPA and sexual maturation in girls | 6-8 y  N = 1,150 | HPLC-MS/MS | 94.7 | Median: 2.0 ng/mL | Median: 2.4 ng/mg creatinine  Range: <LOD-116 ng/mL / <LOD-124 ng/mg creatinine |
| Braun et al., 2011 ^71^  US (Ohio) | To investigate the association between exposure to BPA and behavior/executive function | 1, 2, and 3 y  N = 240 | HPLC-MS/MS | > 97 | Median: 4.1 ng/mL | Median: 14 ng/mg creatinine  IQR: 2.4-7.0 ng/mL / 7.6-21.0 ng/mg creatinine  Range: < LOD -67.0 ng/mL / <LOD-431 ng/mg creatinine |
| Morgan et al., 2011 ^72^  CTEPP study  US (Ohio) | To investigate exposure to BPA and its determinants | 2-5 y  N = 81 | HPLC-MS/MS | 100 | Mean: 8.9 ng/mL  Median: 5.2 ng/mL | Mean: 13.1 ng/mg creatinine  SD: 23.6 ng/mL / 38.7 ng/mg creatinine  Median: 5.9 ng/mg creatinine  IQR: 2.6-7.5 ng/mL / 4-9.1 ng/mg creatinine  GM: 4.8 ng/mL / 6.6 ng/mg creatinine  Range 0.4-211 ng/mL / 0.5-334 ng/mg creatinine |
| Trasande et al., 2012 ^73^  NHANES 2003-2008  US | To investigate the association between exposure to BPA and obesity | 6-19 y  N = 2,838 | HPLC-MS/MS | NR | Median: 2.8 ng/mL | IQR: 1.5-5.6 ng/mL |
| Bhandari et al., 2013 ^74^  NHANES 2003-2008  US | To investigate the association between exposure to BPA and obesity | 6-18 y  N = 2,200 | HPLC-MS/MS | NR | Mean: 4.8 ng/mL | SE: 0.2 ng/mL |
| Donohue et al., 2013 ^75^  US (New York) | To investigate the association between exposure to BPA and asthma development | 3-7 y  N = 408 | HPLC-MS/MS |  | Median  3 y: 3.8 ng/mL  5 y: 3.1 ng/mL  7 y: 2.7 ng/mL | 3 y  IQR: 1.8-7.4 ng/mL  5 y  IQR: 1.7-6.4 ng/mL  7 y  IQR: 1.4-6.0 ng/mL |
| Eng et al., 2013 ^76^  NHANES 2003-2010  US | To investigate the association between exposure to BPA and chronic disease risk factors | 6-18 y  N = 3,370 | HPLC-MS/MS | 95.5 | - | NR |
| Harley et al., 2013 ^77^  US (California) | To investigate the association between exposure to BPA and behavior | 5 y  N = 292 | HPLC-MS/MS | NR | Media: 2.3 ng/mL | Median: 3.2 ng/mg creatinine  IQR: 1.2-4.3 ng/mL / 2.0-5.5 ng/mg creatinine  GM: 2.5 ng/mL / 3.7 ng/mg creatinine  GSD: 3.0 ng/mL / 2.6 ng/mg creatinine  Range: < LOD-442 ng/mL / <LOD-350 ng/mg creatinine |
| Harley et al., 2013 ^78^  CHAMACOS  US (California) | To investigate the association between exposure to BPA and BMI | 5 and 9 y  N = 311 | HPLC-MS/MS | 97.7 (5 y)  89.8 (9 y) | - | 5 y  GM: 2.5 ng/mL  IQR: 1.3-4.6 ng/mL  9 y  GM: 1.6 ng/mL  IQR: 0.9-2.8 ng/mL |
| Hoepner et al., 2013 ^79^  CCCEH study  US (New York) | To investigate exposure to BPA and its determinants | 7 y  N = 318 | HPLC-MS/MS | 96 | Median: 2.7 ng/mL | IQR: 1.4-6.0 ng/mL  GM: 2.9 ng/mL (95%CI: 2.6-3.3) |
| Braun et al., 2014 ^80^  US (Ohio) | To investigate exposure to BPA and its determinants | 1 and 2 y  N = 297 | HPLC-MS/MS |  | Median: 3.6 ng/mL | Median: 13.7 ng/mg creatinine  IQR: 1.8-6.9 ng/mL / 8.4-23.6 ng/mg creatinine |
| Mendonça et al., 2014 ^81^  EARtH  US (Massachusetts) | To investigate exposure to BPA and its determinants | 3-15 mo  N = 29 | HPLC-MS/MS | 93 | Median: 1.8 ng/mL  Mean: 6.0 ng/mL | IQR: 1.2-4.4 ng/mL  SD: 16.2 ng/mL  GM: 2.3 ng/mL (95%CI: 1.5-3.6) |
| Khalil et al., 2014 ^82^  US (Ohio) | To investigate the association between exposure to BPA and cardiometabolic risk factors | 3-8 y  N = 39 | LC-MS/MS | NR | Mean: 1.37 ng/mL | Mean: 1.82 ng/mg creatinine  SD: 2.2 ng/mL / 2.6 ng/mg creatinine |
| Nachman et al., 2015 ^83^  US (Maryland) | To investigate the exposure of neonates to BPA and its temporal trends | Neonates  N = 44 | HPLC-MS/MS | NR | Mean: 0.79 ng/mL  Median: 0.27 ng/mL | SD: 1.7 ng/mL  IQR: <0.1-0.91 ng/mL  Range: < 0.1-11.21 ng/mL |
| Roen et al., 2015 ^84^  US (New York) | To investigate the association between exposure to BPA and behavioral problems | 3-5 y  N = 250 | HPLC-MS/MS | 98 | Mean: 5.2 ng/mL  Median: 3 ng/mL | SD: 6.5 ng/mL  IQR 1.7-6.3 ng/mL  GM: 3.2 ng/mL  Maximum: 54.1 ng/mL |
| Hoepner et al., 2016 ^85^  US (New York) | To investigate the association between exposure to BPA and adiposity | 3 and 5 y  N = 408 (3y)  N = 518 (5y) | HPLC-MS/MS |  | Mean  3y: 8.05 ng/mL  5 y: 5.35 ng/mL | 3 y  SD: 12.53 ng/mL  5 y  SD: 6.51 ng/mL |
| Perera et al., 2016 ^86^  CCCEH study  US (Columbia) | To investigate the association between exposure to BPA and anxiety/depression | 3-5 y  N = 218 | HPLC-MS/MS | 98.6 | Mean: 5.28 ng/mL  Median: 3.05 ng/mL | IQR: 1.8-6.6 ng/mL  GM: 3.29 ng/mL  Range: < LOD-41.2 ng/mL |
| Deierlein et al., 2017 ^87^  BCERC  US (New York) | To investigate the association between exposure to BPA and adiposity | 7 y  N = 1,017 | HPLC-MS/MS | NR | - | NR |
| Kataria et al., 2017 ^88^  US (New York) | To investigate the association between exposure to BPA and oxidant stress/insulin resistance/endothelial dysfunction | 10-13 y  N = 41 | HPLC-MS/MS |  | Median: 0.23 ng/mL | IQR: 0.141-1.16 ng/mL |
| Li et al., 2017 ^89^  NHANES 2003-2006  US | To investigate the association between exposure to BPA and body composition | 8-19 y  N = 1,860 | HPLC-MS/MS | NR | Mean: 5.61 ng/mL | SD: 0.25 ng/mL |
| Stacy et al., 2017 ^90^  US (Ohio) | To investigate the patterns, variability, and determinants of urinary BPA | 1-8 y  N = 337 | HPLC-MS/MS | 94 | Median: 1.6-3.7 ng/mL | Median: 2.1-17.9 ng/mg creatinine (from 1-8y, measured every year) |
| Malits et al., 2018 ^91^  NHANES and CKiD cohort  US | To investigate the association between exposure to BPA and renal function in children with CKD | 1-17 y  N = 538 | HPLC-ESI-MS/MS | NR | Median: 0.78 ng/mL | IQR: 0.18-1.66 ng/mL  GM 0.69 ng/mL (95%CI: 0.61-0.78) |
| Jacobson et al., 2019 ^92^  NHANES 2013-2016  US | To investigate the association between exposure to BPA and obesity | 6-19 y  N = 1,831 | HPLC-MS/MS | 97.5 | Median: 1.3 ng/mL | IQR: 0.7-2.3 ng/mL |
| Liu et al., 2019 ^93^  NHANES 2013-2014  US | To investigate the association between exposure to BPA and obesity | 6-17 y  N = 745 | HPLC-MS/MS | 98.3 | Median: 1.2 ng/mL | IQR: 0.6-2.4 ng/mL |
| Quirós-Alcalá et al., 2021 ^94^  MAACS  US (Baltimore) | To investigate the association between exposure to BPA and asthma morbidity | 5-17 y  N = 148 | HPLC-MS/MS | 100 | Median: 3.4 ng/mL | IQR: 2.2-5.6 ng/mL  GM: 3.6 ng/mL  GSD: 2.2 ng/mL  Range: 0.2-127.5 ng/mL |
| Hu et al., 2022 ^95^  NHANES 2013-2016  US | To investigate the association between exposure to BPA and sex steroid hormones | 6-19 y  N = 1,179 | HPLC-MS/MS | 97.6 | Median: 0.37 ng/mL | IQR: 0.19-0.74 ng/mL  GM 0.52 ng/mL |
| Gajjar et al., 2023 ^96^  US (Ohio) | To investigate the association between exposure to BPA and obesity | 8 y  N = 212 | HPLC-MS/MS | 99.1 | Median: 1.6 ng/mL | IQR: 1.0-3.6 ng/mL |
| Chen et al., 2023 ^97^  NHANES 2007-2012  US | To investigate the association between exposure to BPA and lung function | 6-19 y  N = 1,462 | HPLC-MS/MS | > 93.6 | - | Mean: 3.28 ng/mg creatinine  GM: 2 ng/mg creatinine  Median: 1.83 ng/mg creatinine  IQR: 1.15-3.15 ng/mg creatinine |
| Rocha et al., 2018 ^98^  Brazil | To investigate the association between exposure to BPA and DNA damage | 6-14 y  N = 300 | LC-MS/MS | 98 | Median: 1.66 ng/mL | GM: 1.74 ng/mL  Range: 0.3-35.9 ng/mL |
| Moura et al., 2020 ^99^  Brazil | To validate a method for determining urinary BPA | 6-8 y  N = 343 | HR-MRM | 89.5 | Mean: 3.88 ng/mL  Median: 1.56 ng/mL | Median: 1.64 ng/mg creatinine  Mean: 5.13 ng/mg creatinine  GM: 1.46 ng/mL /1.76 ng/mg creatinine  Range: 0.11-123.0 ng/mL / 0.08-250.6 ng/mg creatinine |
| **Oceania** |  |  |  |  |  |  |
| Heffernan et al., 2014 ^100^  Australia | To investigate exposure to BPA and its determinants | 2-4 y  N = 25 | HPLC-MS/MS | 95 | Median: 2.74 ng/mL | IQR: 1.57-4.68 ng/mL  GM: 2.72 ng/mL  Range: < LOD-74.5 ng/mL |

^a^ Median and/or mean urinary BPA concentrations (ng/mL) when available from individual studies, which were the most frequently reported measures in the included studies.

^b^ Other measures (standard deviation, 95% confidence interval of median values, interquartile range, geometrical mean, and range) when available form individual studies.

95%CI: 95% confidence interval; ADHD: attention-deficit/hyperactivity disorder; APrON: Alberta Pregnancy Outcomes and Nutrition; BCERC: Breast Cancer and the Environment Research Centers; BMI: body mass index; CCCEH: Columbia Center for Children’s Environmental Health; CHAMACOS: Center for the Health Assessment of Mothers and Children of Salinas Cohort; CHMS: Canadian Health Measures Survey; CKD: chronic kidney disease; CKiD: Chronic Kidney Disease in Children. COPHES/DEMOCOPHES: Consortium to Perform Human Biomonitoring on a European Scale / DEMOnstration of a study to COordinate and Perform Human biomonitoring on a European Scale; CTEPP: Assessing the quantitative relationships between preschool children's exposures to bisphenol A by route and urinary biomonitoring; EARtH: Environment and Reproductive Health Study; EDC: Environment and Development of Children; ELEMENT: Early Life Exposures in Mexico to Environmental Toxicants; ESI-MS/MS: electrospray triple quadrupole mass spectrometry; GC-ECNI/MS: gas chromatography-electron capture negative ionization mass spectrometry; GC-MS: gas chromatography- mass spectrometry; GC-MS/MS: gas chromatography-tandem mass spectrometry; GM: geometric mean; GSD: geometric mean standard deviation; GSE: geometric mean standard error; HPLC-ESI-MS/MS: high performance liquid chromatography-electron spray ionization tandem mass spectrometry; HPLC-MS/MS: high-performance liquid chromatography-tandem mass spectrometry; HPLC/FLD: high performance liquid chromatography-fluorescence detection; HR-MRM: liquid chromatography-tandem high-resolution mass spectrometry; ICPP: idiopathic central precocious puberty; ID-LC-MS/MS: isotope dilution-liquid chromatography-tandem mass spectrometry; ICPP: idiopathic central precocious puberty; INMA: Infancia y Medio Ambiente – Environment and Childhood cohort; IQR: interquartile range; KoNEHS: Korean National Environmental Health Survey program; LC-MS: liquid chromatography-mass spectrometry; LC-MS/MS: liquid chromatography-tandem mass spectrometry; LC: liquid chromatography; LC/LC-MS/MS: multidimensional liquid chromatography coupled to tandem mass spectrometry; MAACS: Mouse Allergen and Asthma Cohort Study; NHANES: National Health and Nutritional Examination Survey; REPRO-PL: Polish Mother and Child Cohort Study; SD: standard deviation; SE: standard error; T1D: type 1 diabetes; UPLC-ESI-MS/MS: ultra-performance liquid chromatography-electron spray ionization tandem mass spectrometry; UPLC-MS/MS: ultra-performance liquid chromatography/tandem mass spectrometry; US: United States.

**References**

1 Kim, J. H. *et al.* Bisphenol A-associated epigenomic changes in prepubescent girls: a cross-sectional study in Gharbiah, Egypt. *Environ Health* **12**, 33 (2013).

2 Nahar, M. S. *et al.* Urinary bisphenol A concentrations in girls from rural and urban Egypt: a pilot study. *Environmental Health* **11**, 20 (2012).

3 Youssef, M. M. *et al.* Urinary bisphenol A concentrations in relation to asthma in a sample of Egyptian children. *Hum Exp Toxicol* **37**, 1180-1186 (2018).

4 Wang, H. X. *et al.* Association between bisphenol A exposure and body mass index in Chinese school children: a cross-sectional study. *Environ Health* **11**, 79 (2012).

5 Li, X. *et al.* 4-Nonylphenol, bisphenol-A and triclosan levels in human urine of children and students in China, and the effects of drinking these bottled materials on the levels. *Environ Int* **52**, 81-86 (2013).

6 Wang, B. *et al.* Exposure to bisphenol A among school children in eastern China: a multicenter cross-sectional study. *J Expo Sci Environ Epidemiol* **24**, 657-664 (2014).

7 Zhang, M. *et al.* Occurrence and Profiles of the Artificial Endocrine Disruptor Bisphenol A and Natural Endocrine Disruptor Phytoestrogens in Urine from Children in China. *Int J Environ Res Public Health* **12**, 15110-15117 (2015).

8 Lv, Y. *et al.* Exposure of children to BPA through dust and the association of urinary BPA and triclosan with oxidative stress in Guangzhou, China. *Environ Sci Process Impacts* **18**, 1492-1499 (2016).

9 Chen, Y. *et al.* Association between bisphenol a exposure and idiopathic central precocious puberty (ICPP) among school-aged girls in Shanghai, China. *Environ Int* **115**, 410-416 (2018).

10 Chen, Y. *et al.* Urinary bisphenol analogues and triclosan in children from south China and implications for human exposure. *Environ Pollut* **238**, 299-305 (2018).

11 Li, Y. *et al.* Relationship between bisphenol A exposure and attention-deficit/ hyperactivity disorder: A case-control study for primary school children in Guangzhou, China. *Environ Pollut* **235**, 141-149 (2018).

12 Wang, Z. *et al.* Bisphenol A and pubertal height growth in school-aged children. *J Expo Sci Environ Epidemiol* **29**, 109-117 (2019).

13 Guo, J. *et al.* Urinary bisphenol A concentrations and adiposity measures at age 7 years in a prospective birth cohort. *Chemosphere* **251**, 126340 (2020).

14 Guo, J. *et al.* Maternal and childhood urinary phenol concentrations, neonatal thyroid function, and behavioral problems at 10 years of age: The SMBCS study. *Sci Total Environ* **743**, 140678 (2020).

15 Yang, Y., Shi, Y., Chen, D., Chen, H. & Liu, X. Bisphenol A and its analogues in paired urine and house dust from South China and implications for children's exposure. *Chemosphere* **294**, 133701 (2022).

16 Chen, M. *et al.* Bisphenol A substitutes and childhood obesity at 7 years: a cross-sectional study in Shandong, China. *Environ Sci Pollut Res Int* **30**, 73174-73184 (2023).

17 Xue, J. *et al.* Urinary levels of endocrine-disrupting chemicals, including bisphenols, bisphenol A diglycidyl ethers, benzophenones, parabens, and triclosan in obese and non-obese Indian children. *Environ Res* **137**, 120-128 (2015).

18 Amin, M. M. *et al.* Association of exposure to Bisphenol A with obesity and cardiometabolic risk factors in children and adolescents. *Int J Environ Health Res* **29**, 94-106 (2019).

19 Gys, C. *et al.* Biomonitoring and temporal trends of bisphenols exposure in Japanese school children. *Environ Res* **191**, 110172 (2020).

20 Al-Daghri, N. M. *et al.* BPA exposure is related to metabolic changes in obese Saudi children. *Int J Clin Exp Pathol* **10**, 9910-9916 (2017).

21 Hong, S. B. *et al.* Bisphenol A in relation to behavior and learning of school-age children. *J Child Psychol Psychiatry* **54**, 890-899 (2013).

22 Jung, S. K. *et al.* Profile of Environmental Chemicals in the Korean Population-Results of the Korean National Environmental Health Survey (KoNEHS) Cycle 3, 2015-2017. *Int J Environ Res Public Health* **19** (2022).

23 Choi, J., Eom, J., Kim, J., Lee, S. & Kim, Y. Association between some endocrine-disrupting chemicals and childhood obesity in biological samples of young girls: a cross-sectional study. *Environ Toxicol Pharmacol* **38**, 51-57 (2014).

24 Lim, Y.-H. *et al.* Prenatal and postnatal bisphenol A exposure and social impairment in 4-year-old children. *Environmental Health* **16**, 79 (2017).

25 Jang, Y. *et al.* Associations Between Thyroid Hormone Levels and Urinary Concentrations of Bisphenol A, F, and S in 6-Year-old Children in Korea. *J Prev Med Public Health* **54**, 37-45 (2021).

26 Hwang, M., Choi, K. & Park, C. Urinary levels of phthalate, bisphenol, and paraben and allergic outcomes in children: Korean National Environmental Health Survey 2015-2017. *Sci Total Environ* **818**, 151703 (2022).

27 Kim, B., Park, B., Kim, C. H., Kim, S. & Park, B. Association between endocrine-disrupting chemical mixture and metabolic indices among children, adolescents, and adults: A population-based study in Korea. *Environ Pollut* **315**, 120399 (2022).

28 Kim, J. I. *et al.* Association of bisphenol A, bisphenol F, and bisphenol S with ADHD symptoms in children. *Environ Int* **161**, 107093 (2022).

29 Lee, Y. J. *et al.* Relationship between bisphenol A, bisphenol S, and bisphenol F and serum uric acid concentrations among school-aged children. *PLoS One* **17**, e0268503 (2022).

30 Wang, I. J., Chen, C. Y. & Bornehag, C. G. Bisphenol A exposure may increase the risk of development of atopic disorders in children. *Int J Hyg Environ Health* **219**, 311-316 (2016).

31 Chang, F. K., Shiea, J. & Tsai, H. J. Urinary Concentrations of Triclosan, Benzophenone-3, and Bisphenol A in Taiwanese Children and Adolescents. *Int J Environ Res Public Health* **14** (2017).

32 Lin, T. J., Karmaus, W. J. J., Chen, M. L., Hsu, J. C. & Wang, I. J. Interactions Between Bisphenol A Exposure and GSTP1 Polymorphisms in Childhood Asthma. *Allergy Asthma Immunol Res* **10**, 172-179 (2018).

33 Pornkunwilai, S., Nosoongnoen, W., Jantarat, C., Wachrasindhu, S. & Supornsilchai, V. Urinary bisphenol A detection is significantly associated with young and obese Thai children. *Asian Biomedicine* **9**, 363-372 (2015).

34 Tosirisuk, N. *et al.* Increased bisphenol A levels in Thai children and adolescents with type 1 diabetes mellitus. *Pediatr Int* **64**, e14944 (2022).

35 Çok, İ., İkidağ Ö, T., Battal, D. & Aktaş, A. Assessment of Bisphenol A Levels in Preschool Children: Results of a Human Biomonitoring Study in Ankara, Turkey. *J Clin Res Pediatr Endocrinol* **12**, 86-94 (2020).

36 Covaci, A. *et al.* Urinary BPA measurements in children and mothers from six European member states: Overall results and determinants of exposure. *Environ Res* **141**, 77-85 (2015).

37 Koppen, G. *et al.* Mothers and children are related, even in exposure to chemicals present in common consumer products. *Environ Res* **175**, 297-307 (2019).

38 Frederiksen, H. *et al.* Bisphenol A and other phenols in urine from Danish children and adolescents analyzed by isotope diluted TurboFlow-LC-MS/MS. *Int J Hyg Environ Health* **216**, 710-720 (2013).

39 Becker, K. *et al.* GerES IV: phthalate metabolites and bisphenol A in urine of German children. *Int J Hyg Environ Health* **212**, 685-692 (2009).

40 Kasper-Sonnenberg, M., Koch, H. M., Wittsiepe, J., Brüning, T. & Wilhelm, M. Phthalate metabolites and bisphenol A in urines from German school-aged children: results of the Duisburg birth cohort and Bochum cohort studies. *Int J Hyg Environ Health* **217**, 830-838 (2014).

41 Kasper-Sonnenberg, M., Wittsiepe, J., Wald, K., Koch, H. M. & Wilhelm, M. Pre-pubertal exposure with phthalates and bisphenol A and pubertal development. *PLoS One* **12**, e0187922 (2017).

42 Myridakis, A. *et al.* Phthalate esters, parabens and bisphenol-A exposure among mothers and their children in Greece (Rhea cohort). *Environ Int* **83**, 1-10 (2015).

43 Vafeiadi, M. *et al.* Association of early life exposure to bisphenol A with obesity and cardiometabolic traits in childhood. *Environ Res* **146**, 379-387 (2016).

44 Nicolucci, C. *et al.* A high selective and sensitive liquid chromatography-tandem mass spectrometry method for quantization of BPA urinary levels in children. *Anal Bioanal Chem* **405**, 9139-9148 (2013).

45 D'Aniello, R. *et al.* Emerging pathomechanisms involved in obesity. *J Pediatr Gastroenterol Nutr* **60**, 113-119 (2015).

46 Bellisario, V. *et al.* Bisphenol A and S in the Urine of Newborns: Plastic for Non-Food Use Still without Rules. *Biology (Basel)* **10** (2021).

47 Tait, S. *et al.* Italian Children Exposure to Bisphenol A: Biomonitoring Data from the LIFE PERSUADED Project. *Int J Environ Res Public Health* **18** (2021).

48 Sakhi, A. K., Sabaredzovic, A., Papadopoulou, E., Cequier, E. & Thomsen, C. Levels, variability and determinants of environmental phenols in pairs of Norwegian mothers and children. *Environ Int* **114**, 242-251 (2018).

49 Garí, M. *et al.* Human-Biomonitoring derived exposure and Daily Intakes of Bisphenol A and their associations with neurodevelopmental outcomes among children of the Polish Mother and Child Cohort Study. *Environmental Health* **20**, 95 (2021).

50 Correia-Sá, L. *et al.* Exposure assessment to bisphenol A (BPA) in Portuguese children by human biomonitoring. *Environ Sci Pollut Res Int* **24**, 27502-27514 (2017).

51 Casas, L. *et al.* Urinary concentrations of phthalates and phenols in a population of Spanish pregnant women and children. *Environment International* **37**, 858-866 (2011).

52 Casas, M. *et al.* Dietary and sociodemographic determinants of bisphenol A urine concentrations in pregnant women and children. *Environ Int* **56**, 10-18 (2013).

53 Cutanda, F. *et al.* Urinary levels of eight phthalate metabolites and bisphenol A in mother–child pairs from two Spanish locations. *International Journal of Hygiene and Environmental Health* **218**, 47-57 (2015).

54 Perez-Lobato, R. *et al.* Exposure to bisphenol A and behavior in school-age children. *Neurotoxicology* **53**, 12-19 (2016).

55 Mustieles, V. *et al.* Bisphenol A and reproductive hormones and cortisol in peripubertal boys: The INMA-Granada cohort. *Sci Total Environ* **618**, 1046-1053 (2018).

56 Mustieles, V. *et al.* Bisphenol A and adiposity measures in peripubertal boys from the INMA-Granada cohort. *Environ Res* **173**, 443-451 (2019).

57 Snoj Tratnik, J. *et al.* Urinary bisphenol A in children, mothers and fathers from Slovenia: Overall results and determinants of exposure. *Environ Res* **168**, 32-40 (2019).

58 Larsson, K. *et al.* Exposure determinants of phthalates, parabens, bisphenol A and triclosan in Swedish mothers and their children. *Environ Int* **73**, 323-333 (2014).

59 Arbuckle, T. E., Davis, K., Boylan, K., Fisher, M. & Fu, J. Bisphenol A, phthalates and lead and learning and behavioral problems in Canadian children 6-11 years of age: CHMS 2007-2009. *Neurotoxicology* **54**, 89-98 (2016).

60 England-Mason, G. *et al.* Postnatal BPA is associated with increasing executive function difficulties in preschool children. *Pediatr Res* **89**, 686-693 (2021).

61 Findlay, L. C. & Kohen, D. E. Bisphenol A and child and youth behaviour: Canadian Health Measures Survey 2007 to 2011. *Health Rep* **26**, 3-9 (2015).

62 Grohs, M. N. *et al.* Prenatal maternal and childhood bisphenol a exposure and brain structure and behavior of young children. *Environ Health* **18**, 85 (2019).

63 Jacobson, M. H. *et al.* Serially assessed bisphenol A and phthalate exposure and association with kidney function in children with chronic kidney disease in the US and Canada: A longitudinal cohort study. *PLoS Med* **17**, e1003384 (2020).

64 Lewis, R. C. *et al.* Predictors of urinary bisphenol A and phthalate metabolite concentrations in Mexican children. *Chemosphere* **93**, 2390-2398 (2013).

65 Ferguson, K. K. *et al.* Prenatal and peripubertal phthalates and bisphenol A in relation to sex hormones and puberty in boys. *Reprod Toxicol* **47**, 70-76 (2014).

66 Watkins, D. J. *et al.* In utero and peripubertal exposure to phthalates and BPA in relation to female sexual maturation. *Environ Res* **134**, 233-241 (2014).

67 Calafat, A. M., Ye, X., Wong, L. Y., Reidy, J. A. & Needham, L. L. Exposure of the U.S. population to bisphenol A and 4-tertiary-octylphenol: 2003-2004. *Environ Health Perspect* **116**, 39-44 (2008).

68 Teitelbaum, S. L. *et al.* Temporal variability in urinary concentrations of phthalate metabolites, phytoestrogens and phenols among minority children in the United States. *Environ Res* **106**, 257-269 (2008).

69 Wolff, M. S. *et al.* Environmental exposures and puberty in inner-city girls. *Environ Res* **107**, 393-400 (2008).

70 Wolff, M. S. *et al.* Investigation of relationships between urinary biomarkers of phytoestrogens, phthalates, and phenols and pubertal stages in girls. *Environ Health Perspect* **118**, 1039-1046 (2010).

71 Braun, J. M. *et al.* Impact of early-life bisphenol A exposure on behavior and executive function in children. *Pediatrics* **128**, 873-882 (2011).

72 Morgan, M. K. *et al.* Assessing the quantitative relationships between preschool children's exposures to bisphenol A by route and urinary biomonitoring. *Environ Sci Technol* **45**, 5309-5316 (2011).

73 Trasande, L., Attina, T. M. & Blustein, J. Association between urinary bisphenol A concentration and obesity prevalence in children and adolescents. *Jama* **308**, 1113-1121 (2012).

74 Bhandari, R., Xiao, J. & Shankar, A. Urinary bisphenol A and obesity in U.S. children. *Am J Epidemiol* **177**, 1263-1270 (2013).

75 Donohue, K. M. *et al.* Prenatal and postnatal bisphenol A exposure and asthma development among inner-city children. *J Allergy Clin Immunol* **131**, 736-742 (2013).

76 Eng, D. S. *et al.* Bisphenol A and chronic disease risk factors in US children. *Pediatrics* **132**, e637-645 (2013).

77 Harley, K. G. *et al.* Prenatal and early childhood bisphenol A concentrations and behavior in school-aged children. *Environ Res* **126**, 43-50 (2013).

78 Harley, K. G. *et al.* Prenatal and postnatal bisphenol A exposure and body mass index in childhood in the CHAMACOS cohort. *Environ Health Perspect* **121**, 514-520 (2013).

79 Hoepner, L. A. *et al.* Urinary concentrations of bisphenol A in an urban minority birth cohort in New York City, prenatal through age 7 years. *Environ Res* **122**, 38-44 (2013).

80 Braun, J. M. *et al.* Early-life bisphenol a exposure and child body mass index: a prospective cohort study. *Environ Health Perspect* **122**, 1239-1245 (2014).

81 Mendonca, K., Hauser, R., Calafat, A. M., Arbuckle, T. E. & Duty, S. M. Bisphenol A concentrations in maternal breast milk and infant urine. *Int Arch Occup Environ Health* **87**, 13-20 (2014).

82 Khalil, N. *et al.* Bisphenol A and cardiometabolic risk factors in obese children. *Sci Total Environ* **470-471**, 726-732 (2014).

83 Nachman, R. M. *et al.* Serial Free Bisphenol A and Bisphenol A Glucuronide Concentrations in Neonates. *J Pediatr* **167**, 64-69 (2015).

84 Roen, E. L. *et al.* Bisphenol A exposure and behavioral problems among inner city children at 7-9 years of age. *Environ Res* **142**, 739-745 (2015).

85 Hoepner, L. A. *et al.* Bisphenol A and Adiposity in an Inner-City Birth Cohort. *Environ Health Perspect* **124**, 1644-1650 (2016).

86 Perera, F. *et al.* Bisphenol A exposure and symptoms of anxiety and depression among inner city children at 10-12 years of age. *Environ Res* **151**, 195-202 (2016).

87 Deierlein, A. L. *et al.* Phenol Concentrations During Childhood and Subsequent Measures of Adiposity Among Young Girls. *Am J Epidemiol* **186**, 581-592 (2017).

88 Kataria, A. *et al.* Exposure to bisphenols and phthalates and association with oxidant stress, insulin resistance, and endothelial dysfunction in children. *Pediatr Res* **81**, 857-864 (2017).

89 Li, J., Lai, H., Chen, S., Zhu, H. & Lai, S. Gender differences in the associations between urinary bisphenol A and body composition among American children: The National Health and Nutrition Examination Survey, 2003-2006. *J Epidemiol* **27**, 228-234 (2017).

90 Stacy, S. L. *et al.* Patterns, Variability, and Predictors of Urinary Bisphenol A Concentrations during Childhood. *Environ Sci Technol* **50**, 5981-5990 (2016).

91 Malits, J. *et al.* Renal Function and exposure to Bisphenol A and phthalates in children with Chronic Kidney Disease. *Environ Res* **167**, 575-582 (2018).

92 Jacobson, M. H., Woodward, M., Bao, W., Liu, B. & Trasande, L. Urinary Bisphenols and Obesity Prevalence Among U.S. Children and Adolescents. *J Endocr Soc* **3**, 1715-1726 (2019).

93 Liu, B. *et al.* Association of Bisphenol A and Its Substitutes, Bisphenol F and Bisphenol S, with Obesity in United States Children and Adolescents. *Diabetes Metab J* **43**, 59-75 (2019).

94 Quirós-Alcalá, L. *et al.* Exposure to bisphenols and asthma morbidity among low-income urban children with asthma. *J Allergy Clin Immunol* **147**, 577-586.e577 (2021).

95 Hu, P. *et al.* Associations between exposure to a mixture of phenols, parabens, and phthalates and sex steroid hormones in children 6-19 years from NHANES, 2013-2016. *Sci Total Environ* **822**, 153548 (2022).

96 Gajjar, P. *et al.* Associations of mid-childhood bisphenol A and bisphenol S exposure with mid-childhood and adolescent obesity. *Environ Epidemiol* **6**, e187 (2022).

97 Chen, Y. *et al.* Individual and joint association of phenols, parabens, and phthalates with childhood lung function: Exploring the mediating role of peripheral immune responses. *J Hazard Mater* **454**, 131457 (2023).

98 Rocha, B. A. *et al.* Advanced data mining approaches in the assessment of urinary concentrations of bisphenols, chlorophenols, parabens and benzophenones in Brazilian children and their association to DNA damage. *Environ Int* **116**, 269-277 (2018).

99 Moura, H. S. R. P., Rocha, P. R. S., Amato, A. A. & Sodré, F. F. Quantification of bisphenol A in urine samples from children studying in public schools from the Brazilian Capital. *Microchemical Journal* **152**, 104347 (2020).

100 Heffernan, A. L., Sly, P. D., Toms, L. M., Hobson, P. & Mueller, J. F. Bisphenol A exposure is not associated with area-level socioeconomic index in Australian children using pooled urine samples. *Environ Sci Pollut Res Int* **21**, 9344-9355 (2014).
